# Nanotechnology for Targeted Therapy of Atherosclerosis

**DOI:** 10.3389/fphar.2021.755569

**Published:** 2021-11-12

**Authors:** Seyedmehdi Hossaini Nasr, Xuefei Huang

**Affiliations:** ^1^ Department of Chemistry, Michigan State University, East Lansing, MI, United States; ^2^ Institute for Quantitative Health Science and Engineering, Michigan State University, East Lansing, MI, United States; ^3^ Department of Biomedical Engineering, Michigan State University, East Lansing, MI, United States

**Keywords:** atherosclerosis, nanomedicine, drug delivery, inflammation, statins

## Abstract

Atherosclerosis is the major cause of heart attack and stroke that are the leading causes of death in the world. Nanomedicine is a powerful tool that can be engineered to target atherosclerotic plaques for therapeutic and diagnosis purposes. In this review, advances in designing nanoparticles with therapeutic effects on atherosclerotic plaques known as atheroprotective nanomedicine have been summarized to stimulate further development and future translation.

## Introduction

Atherosclerosis is the thickening of artery vessel walls that can develop in arteries supplying blood to various organs such as heart, brain, and kidneys. It is a major cause of heart attack and stroke (Weber and Noels, 2011), the leading causes of death in the world. The majority of patients who experience cardiac arrests have atherosclerosis (Chelly et al., 2012). It has been estimated that by 2030, the global cost of cardiovascular diseases would reach a staggering number, $1,044 billion (Benjamin et al., 2017).

**FIGURE 1:**
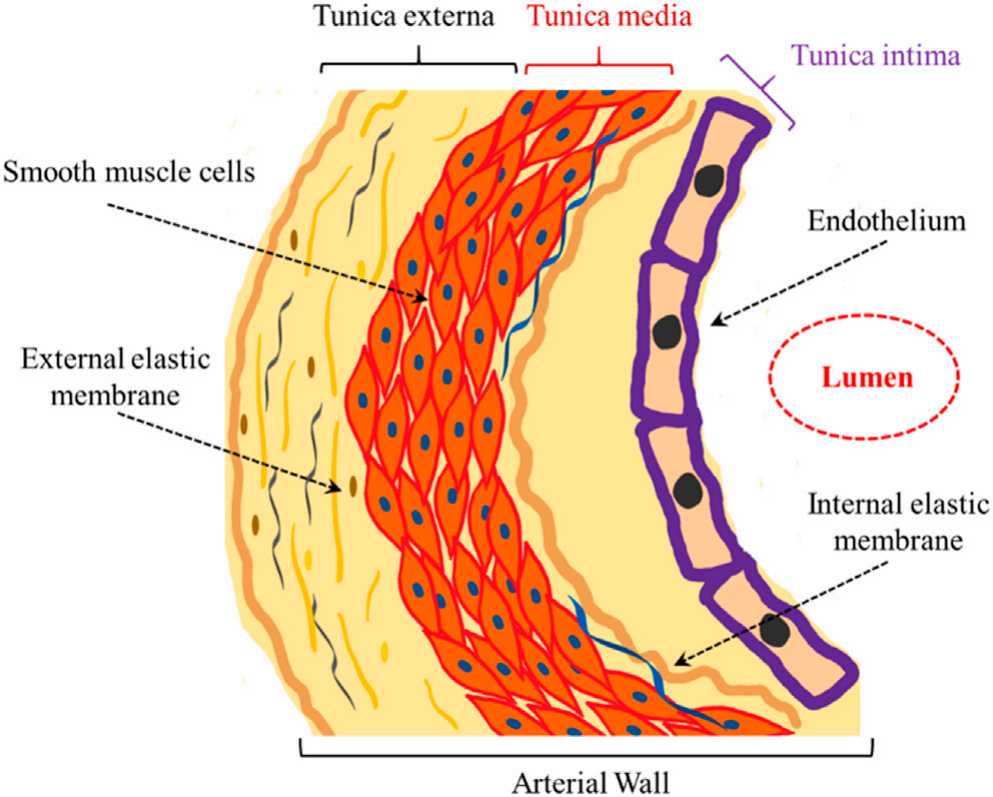
Schematic demonstration of the arterial wall structure.

To better understand atherosclerosis pathology, it is important to know the structure of arterial walls, which consist of three different parts, i.e., tunica intima, tunica media and tunica externa (Figure 1). Squamous endothelial cells line the tunica intima and internal elastic lamina separates it from the tunica media. The tunica media contains elastic lamellae and elastic fibers alternating with layers of smooth muscle cells. Proteoglycans and reticular fibers are the other components of the media. Finally, connective tissues consisting of collagen and elastic fibers form the tunica externa (Mescher, 2016).

Atherosclerosis is a chronic inflammatory condition (Weber and Noels, 2011). One important trigger for the formation of atherosclerotic plaques is hypercholesterolemia. Cholesterol is transported in the plasma through lipoproteins. Among them, apolipoprotein B is responsible for carrying cholesterol and fat molecule around the body generally packed in particles known as low density lipoprotein (LDL). High levels of cholesterol in plasma results in the accumulation of lipids into the arterial wall. This accrual is mediated mostly through LDL particles together with changes in endothelial cell permeabilities. Overexpression of receptors such as vascular adhesion molecule 1 (VCAM-1) and selectins enhances their interactions with LDL and mediates LDL entrance into the arterial wall (Sakakura et al., 2013). In addition, oxidized LDL (oxLDL) favors the intracellular accumulation of cholesterol esters (Maiolino et al., 2013). Moreover, oxLDL is a potent inducer of inflammatory molecules and mediates monocyte binding to endothelial cells (Berliner et al., 1995).

Both innate and adaptive immunity play roles in pathophysiology of atherosclerosis (Herrero-Fernandez et al., 2019). As mentioned above, monocytes and macrophages, as the main contributors of innate immune response, initiate the progression of atherosclerosis. Following this step, the adaptive immune system can get activated and promote the plaque formation (Miteva et al., 2018). Type 1 helper T (Th1) cells, type 2 helper T (Th2) cells, regulatory T (Treg) cells, CD4^+^ T cells and CD8^+^ T cells are the main contributors of the adaptive immune system involved in plaque formation and resolution (Roy et al., 2021). The understanding of the mechanisms of immune responses involved in pathophysiology of inflammatory atherosclerosis not only bring in a realistic perception of the complexity of the disease but also inspire the development of novel therapeutics (Ridker, 2021).

**GRAPHICAL ABSTRACT:**
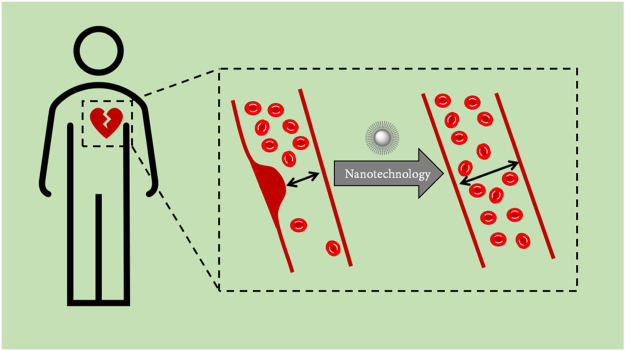


## Current Atherosclerotic Treatment Methods

Management of atherosclerosis typically involves drugs and/or surgery. Surgical intervention includes procedures such as angioplasty and bypass grafting. In angioplasty, a blocked or narrowed artery can be reopened and sometimes a physical device called stent is placed there to keep it open (Derdeyn and Chimowitz, 2007), while in bypass grafting, a healthy blood vessel is used to circumvent the area of atherosclerosis plaques (Martínez-González et al., 2017).

For pharmacological interventions, statins are one of the most important classes. Statins inhibit 3-hydroxy-3-methylglutaryl coenzyme A (HMG-CoA) reductase, which is a key enzyme for cholesterol biosynthesis (Cerqueira et al., 2016). Since a high cholesterol level in blood is an important risk factor for cardiovascular diseases (CVD), statins are believed to reduce possible CVD events through reduction of cholesterol (Taylor et al., 2013). In addition to their cholesterol modulating activities, statins can exert cardiovascular protecting effects through other pathways (Oesterle et al., 2017). These pleiotropic effects are mostly the result of isoprenylation inhibition that reduces the function of isoprenoids on targets such as Rho and Rac proteins. These proteins are involved in the expression of proinflammatory cytokines and transduction of signaling molecules. For instance, they can modulate reactive oxygen species (ROS) generation and their reduction can result in anti-inflammatory outcomes (Wolfrum et al., 2003). An important study that addressed anti-inflammatory effect of statins independent of their effect on LDL cholesterol level was conducted by Ridker and coworkers. In this study, Pravastatin was shown to reduce C-reactive protein (CRP) levels in people when assessed 12 and 24 weeks after taking the drug (Albert et al., 2001). CRP is known as a sensitive systemic marker for inflammation (Pepys and Baltz, 1983) and any acute inflammatory stimulus may cause a rapid rise of CRP level up to 1,000 folds (Black et al., 2004). Besides CRP, statins can reduce expression of cyclooxygenase-2 (Cox-2) in human endothelial cells (Massaro et al., 2010), murine macrophages (RAW264.7) (Shao et al., 2012) and atherosclerosis lesions in rabbits (Hernandez-Presa et al., 2002). Cox-2 is an inducible pro-inflammatory enzyme, which is mainly present (Gately and Li, 2004) in inflammatory macrophages in atherosclerotic plaques (Burleigh et al., 2005). Pro-inflammatory macrophages or M1 are known to propagate proinflammatory cytokines such as tumor necrosis factor-α (TNFα), interleukin (IL)-1 and IL-6 (Autieri, 2012). Statins can also alleviate proinflammatory cytokines effects. For instance, reduction of TNFα, IL-1 and IL-6 have been observed in hypercholesterolemic patients who received atorvastatin for 2 months (Ascer et al., 2004). High dose statin therapy can lead to the rapid reduction of atherosclerosis inflammation (Nissen et al., 2006; Tawakol et al., 2013) indicating the potential of this medicine for management of inflammatory atherosclerotic plaques. While statins have shown their beneficial effects, the therapeutic regimen should be carefully designed to prevent undesired outcomes. For example, discontinuation of statins results in rebound inflammation and increased short-term cardiovascular risks, even in the absence of changes in lipid levels (Heeschen et al., 2002; Spencer et al., 2004).

Besides statins, there are other therapeutic molecules for management of atherosclerosis. Proprotein convertase subtilisin/kexin type 9 (PCSK9) is an enzyme involved in LDL homeostasis. Agents that can block PCSK9 can enhance LDL degradation and lower cholesterol levels. Two PCSK9 inhibitors, alirocumab and evolocumab, were approved by the U.S. Food and Drug Administration to be used in patients not tolerant of statins. In addition, anti-inflammatory agents are entering the clinic for atherosclerosis complications. For instance, canakinumab, which targets human IL-1β, has shown promising results when evaluated in patients with CVD complications and high levels of CRP (Ridker et al., 2017). In addition, immunosuppressants such as methotrexate and rapamycin have shown promising effects to reduce atherosclerotic plaque sizes when studied in ApoE knockout mice (Zhang X. et al., 2017; Stigliano et al., 2017).

The incorporation of nanotechnology into atherosclerosis management is an exciting direction to pursue complementing the current treatment methods (Peters et al., 2021). While anti-inflammatory approach is a popular drug therapy regimen, the choice of suitable anti-inflammatory molecules requires consideration. Prednisolone, a corticosteroid drug, showed high accumulation in plaque macrophages when formulated in liposomal nanoparticle (LN-PLP) (Lobatto et al., 2010). However, further studies ruled out this medicine for atherosclerosis management. *In vitro* experiments showed lipotoxic effects of LN-PLP when incubated with macrophages (Van Der Valk et al., 2016). *In vivo* analysis indicated the drug accelerated atherosclerosis increasing the size of the necrotic areas. Another example is a study that compared the effect of anti-proliferative drugs used in vascular restenosis treatment (Chen et al., 2021). Although sirolimus and paclitaxel nanoparticles both exert anti-proliferation effects under normoxia conditions, sirolimus nanoparticle showed a significantly higher effect under the hypoxia condition. Therefore, sirolimus nanoparticle would be a better candidate for atherosclerosis treatment due to the presence of hypoxic microenvironment in the plaques. These results highlight the importance of drug choice in treatment of atherosclerosis.

## Strategies for Active Targeting of Atherosclerosis

### Active Targeting of Macrophages

**FIGURE 2:**
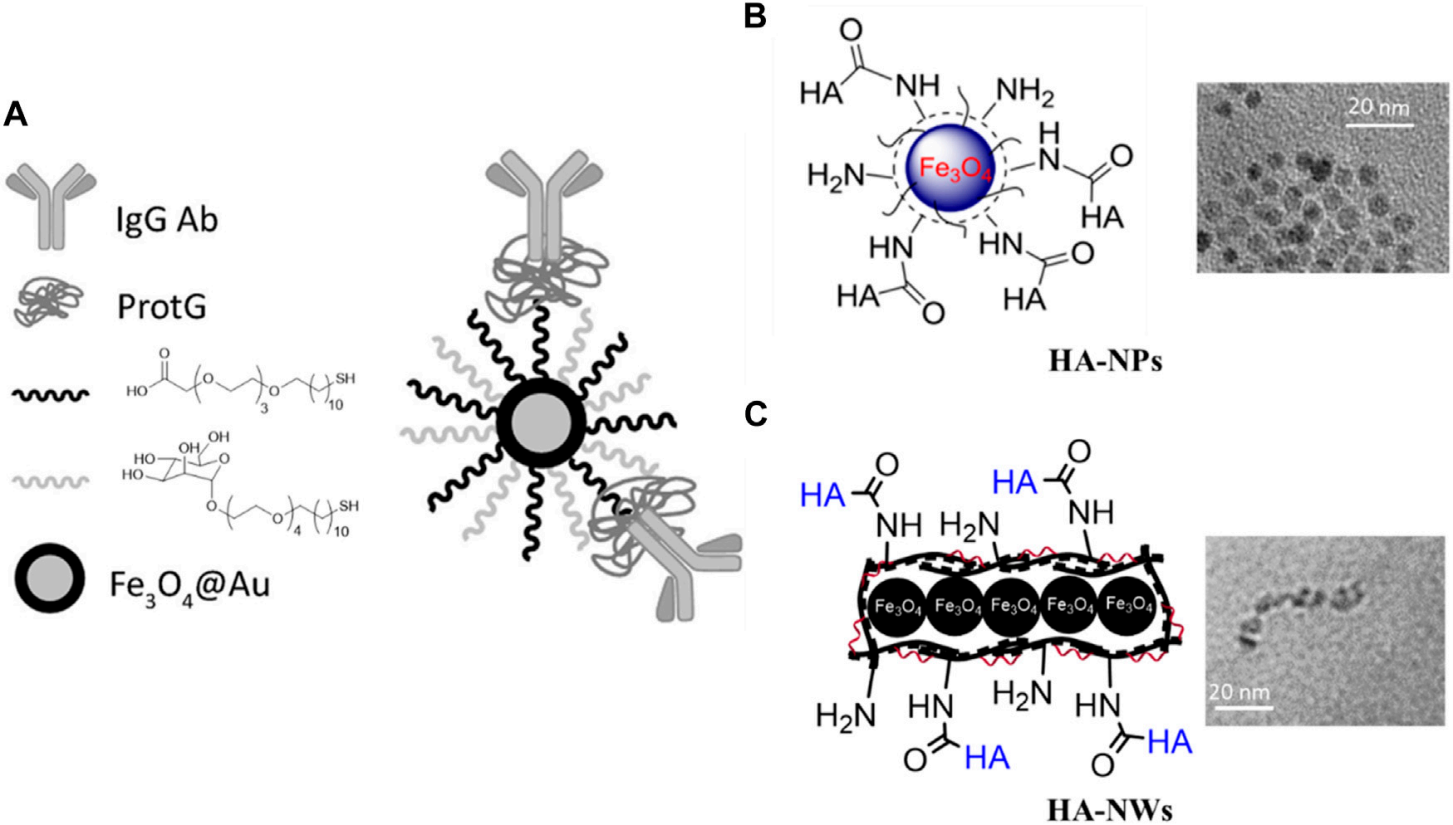
Iron oxide nanoparticles for active targeting of atherosclerotic plaques. **(A)** Antibody directed nanoparticle for atherosclerotic plaque imaging. Gold coated iron oxide nanoparticles were first functionalized with mannose and carboxylic acid ending ligand, then protG was immobilized on nanoparticles. Finally, the nanoparticles were decorated with an anti-CD163 antibody. These nanoparticles were used for MRI and CT imaging of plaques (Tarin et al., 2015) (Copyright permission from Elsevier). **(B)** HA conjugated iron oxide NPs. These MRI probes were targeted towards the CD44 receptor for imaging of atherosclerotic plaques (El-Dakdouki et al., 2014). **(C)** HA conjugated worm shaped iron oxide nanoprobes. These HA-NWs induced lower inflammatory responses compared to the corresponding HA-NPs with a spherical morphology (Hossaini Nasr et al., 2018) (Copyright permission from American Chemical Society).

As mentioned earlier, macrophages and monocytes play important roles in the formation and progression of atherosclerosis. Not surprisingly, these cells have been considered extensively for imaging and/or therapeutic purposes of atherosclerosis. The phagocytic activities of macrophages together with their abilities to traffic into the inflammatory sites make them a good candidate for targeting atherosclerosis plaques (Kircher et al., 2008). Methods enabling active targeting of inflammatory macrophages can provide additional advantages. For example, while 500–1,000 μmol Fe/kg body weight was needed for magnetic resonance imaging (MRI) of atherosclerosis plaques in ApoE knockout mouse through non-targeted iron oxide nanoparticles (Klug et al., 2009), the amounts of agents needed could be reduced to 36 μmol Fe/kg when iron oxide nanoprobes were decorated with anti-CD163 mAb for active targeting of macrophages residing in inflammatory atherosclerotic plaques (Figure 2A) (Tarin et al., 2015). Moreover, polysaccharide hyaluronic acid (HA) conjugated iron oxide nanoparticles (HA-NP) (El-Dakdouki et al., 2014) have been developed for active targeting of macrophage CD44 receptor enabling *in vivo* imaging of atherosclerotic plaques in a rabbit model of atherosclerosis following injection of 3.75 μmol Fe/kg (Figure 2B). Other studies used at least 10 times higher amount of Fe/kg for imaging atherosclerosis plaques in this animal model without installing a recognition element on the nanoparticles capable of binding with a receptor on the surface of macrophages (Yu et al., 2011; Tsuchiya et al., 2013). Moreover, active targeting of atherosclerotic plaques by HA bearing iron oxide nanoparticles can be improved using worm shape morphology. For instance, HA conjugated iron oxide nanoworms (HA-NW) (Hossaini Nasr et al., 2018) showed a higher binding with CD44 expressing cells *in vitro* and induced lower inflammatory responses *in vivo*, when administered to ApoE knockout mice (Figure 2C).

**FIGURE 3:**
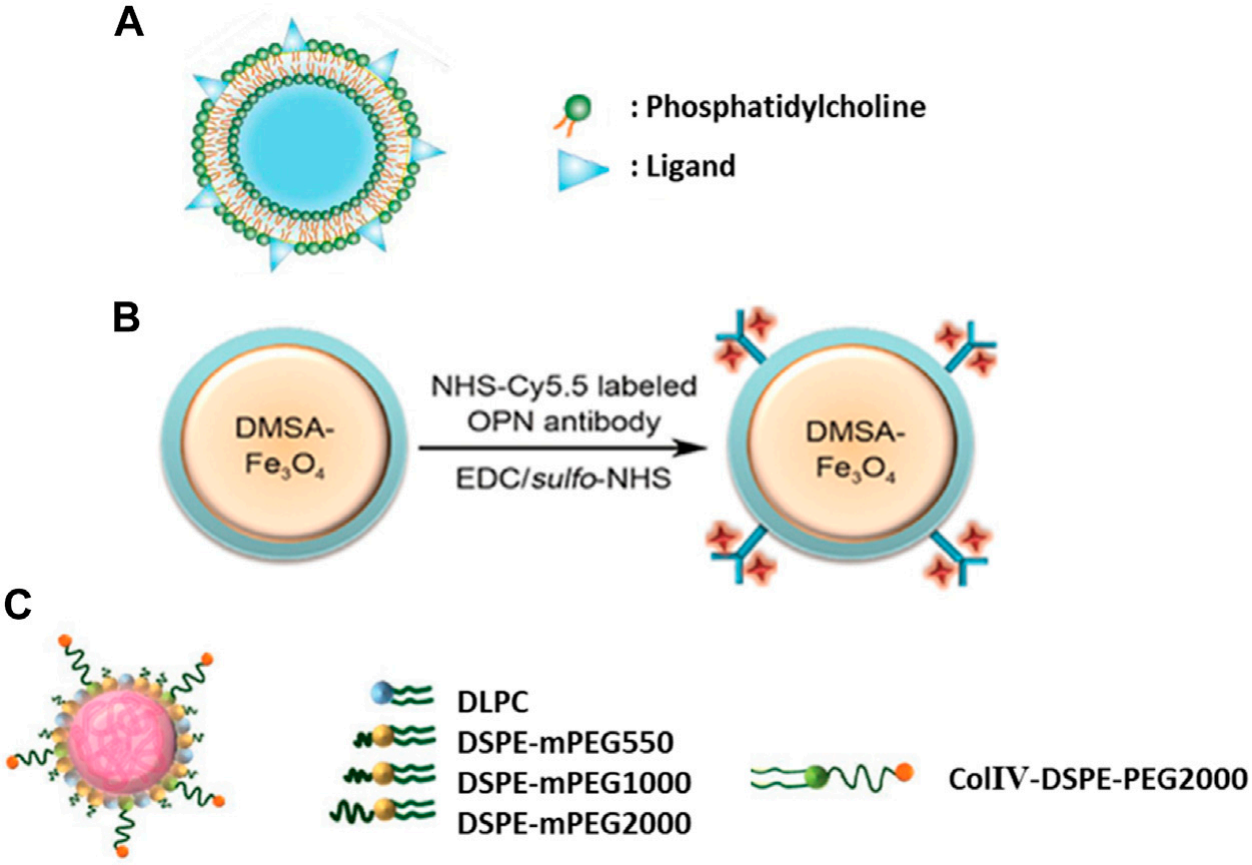
**(A)** Liposome-like nanovesicle was prepared to target CD36 receptor through KOdiA-PC ligand (Copyright permission from Elsevier). **(B)** Iron oxide nanoparticles were conjugated with an anti-osteopontin antibody labeled with Cy5.5 fluorescent dye for dual modality imaging of plaques (Copyright permission from Elsevier). **(C)** Lipid-polymer hybrid nanoparticles contained three components: a biodegradable hydrophobic polymeric core, a lipid layer and a peptide decoration for collagen IV (ColIV) targeting (Copyright permission from John Wiley & Sons).

Besides antibodies and carbohydrates, active targeting of atherosclerotic plaques has been studied through various other strategies. For instance, activated macrophages and foam cells residing in inflammatory atherosclerosis plaques overexpress myeloperoxidase (MPO) (Daugherty et al., 1994). Gd based MPO sensor was designed to detect inflammation in a rabbit model of atherosclerosis (Ronald et al., 2009). Another approach for active targeting of macrophages was the coating of nanocarrier with oxidized phosphatidylcholine (Nie et al., 2015). Interaction of macrophages with oxidized phosphatidylcholine [e.g., 1-(palmitoyl)-2-(5-keto-6-octene-dioyl)] through CD36 receptor has been explored (Collot-Teixeira et al., 2007; Seimon et al., 2010), due to the ability of the oxidized lipid to bind with CD36 (IC50: 3.9 μM) (Figure 3A) (Podrez et al., 2002). Another example is a cytokine known as osteopontin (OPN) expressed by foamy macrophages to recruit leukocytes and induce matrix metalloproteinase expression (Matsui et al., 2003; Scatena et al., 2007). An anti-OPN mAb has been used for imaging vulnerable atherosclerotic plaques with various engineered nanoprobes (Figure 3B) (Qiao H. et al., 2017; Qiao R. et al., 2017).

An additional molecule for targeting apoptotic macrophages in atherosclerotic plaques is annexin v (Sarai et al., 2007). Decoration of nanoprobes with annexin v protein can target phosphatidylserine present on membrane of apoptotic macrophages in atherosclerotic plaques (Li et al., 2016). Interestingly, virus like particles have also been used for targeting macrophages. For instance, Simian virus 40 (SV40) has been loaded with imaging and therapeutic molecules and targeted to p32 protein on macrophage surface through insertion of CGNKRTRGC peptide known as LyP-1 (Hamzah et al., 2011). In addition, a therapeutic peptide, Hirulog, has been incorporated, which is a thrombin inhibitor with anti-coagulant activities (Sun et al., 2016). Elaborate protein engineering was applied to insert the peptide to guide targeting with the Hirulog peptide pointing in after self-assembly of SV40 virus nanoparticles.

Physicochemical properties of a nanomedicine can be elaborately adjusted to exert atheroprotective effects. For instance, Lewis and coworkers synthesized nanoparticles that exhibited significant targeting and therapeutic effects when tested in ApoE knockout mice (Lewis et al., 2015). A library of composite nanoparticles with different shells consisted of sugar-based amphiphilic macromolecules (AMs) were prepared. Interestingly, the fully synthetic AM constructs could bind with the scavenger receptors on atherogenic macrophages through mimicking physicochemical properties (charge and hydrophobicity) of oxidized lipoproteins (Chnari et al., 2006) resulting in their accumulation in the plaques. They also competed with oxLDL uptake and downregulated the expression of primary scavenger receptors such as CD36 and macrophage scavenger receptor 1 (MSR1) to exert their atheroprotective effects for a site-directed treatment for atherosclerosis plaques.

### Active Targeting Through Adhesion Molecules

Angiogenesis targeting is another strategy for imaging atherosclerotic lesions. Since integrin molecules such as αvβ3 play critical roles in angiogenesis, they could be targeted by an integrin receptor antagonist (Winter et al., 2003). In addition, endothelial cells over-express adhesion molecules such as vascular cell adhesion molecule-1 (VCAM-1), intracellular adhesion molecule-1 (ICAM-1) and selectins in atherosclerotic plaques (Khodabandehlou et al., 2017). Thus, various studies have targeted adhesion molecules to direct nanoprobes. For instance, VCAM-1 has been targeted by a short peptide (VHPKQHRAEEAK) (Bruckman et al., 2014) to image atherosclerotic plaques in ApoE knockout mice. Anti-ICAM-1 mAb is another approach for targeting endothelial cell adhesion molecules and delivering their payload through cell adhesion molecule-mediated endocytosis into the plaque site (Serrano et al., 2012). In other studies, microparticles containing atheroprotective microRNA (miR-146a/-181b) have been targeted to E-selectin through a thioaptamer molecule (Ma et al., 2016).

### Active Targeting of Extracellular Matrix

Collagen is an important target for atherosclerotic lesions especially since collagen IV forms the bases of blood vessels and it gets exposed during atherosclerosis progression (Murata et al., 1986). Phage display has been used to discover short peptides enabling the targeting of collagen IV (Chan et al., 2010). Theranostic nanoparticles have been engineered to deliver their payload into the atherosclerosis plaques through collagen IV binding peptide (Figure 3C) (Fredman et al., 2015; Kamaly et al., 2016; Yu et al., 2017). Another protein molecule for atherosclerosis targeting is profilin-1, which is an actin binding protein (Romeo et al., 2007). The over-expression of profilin-1 is associated with atherosclerosis and other cardiovascular complications. Subsequently, nanoparticles have been conjugated with anti-profilin-1 molecule to deliver therapeutics such as profilin-1 siRNA into the plaque sites. Profilin-1 silencing exerts its therapeutic properties by reducing mouse aorta smooth muscle cell proliferation and migration (Wang et al., 2016).

## HDL Like Nanoparticles for Drug Delivery to Atherosclerotic Plaques

**FIGURE 4:**
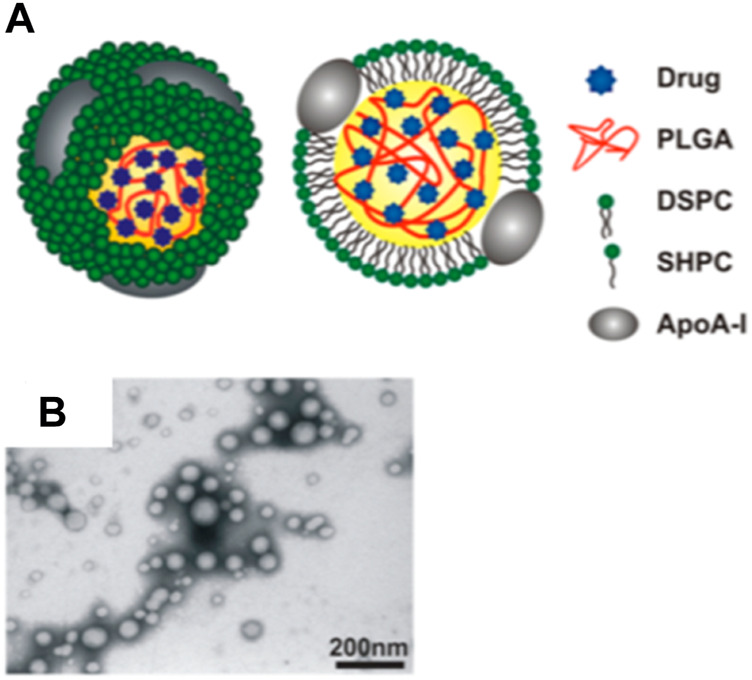
HDL like nanoparticles. **(A)** Schematic demonstration of the PLGA-HDL nanoparticles with PLGA forming the polymeric core of this nanoparticle; **(B)** TEM images showed the spherical morphology of the nanoparticles (Copyright permission from the American Chemical Society).

In human bodies, two endogenous nanoparticles known as high-density lipoprotein (HDL) and LDL, play important roles in managing cholesterol homeostasis. LDL are small carriers of fat molecules to the cells, which can penetrate into arterial walls, get oxidized and cause inflammation. In contrast, HDL can interact with lipid-laden plaque macrophages, and mediate cholesterol removal from plaques by transporting them into the liver (Wang et al., 2004; Duong et al., 2006). The diameter of HDL is about 8–12 nm and hydrophobic lipids are located in the core while phospholipids and apolipoproteins such as apolipoprotein A (ApoA)-1 or ApoA-II form the outer layer (Gordon et al., 2010). Based on the knowledge of HDL structures and functions, scientists have investigated HDL and/or HDL mimetic nanoparticles to target atherosclerotic plaques. For instance, microfluidic technology has been used to build poly (lactic-co-glycolic acid) (PLGA)-HDL nanocarrier (Sanchez-Gaytan et al., 2015). This nanocarrier contains a PLGA core capable of loading hydrophobic drugs while it is decorated with ApoA-1 and phospholipids such as 1,2-distearoyl-sn-glycero-3-phosphocholine and 1-stearoyl-2-hydroxy-sn-glycero-3-phosphocholine (Figure 4). PLGA-HDL showed a slow drug release behavior with 60% release in 24 h and up to 90% release after 5 days in PBS buffer. Moreover, animal study on ApoE knockout mice showed 12.8-h half-life in blood circulation and their targeting property was confirmed by high accumulation of these particles in macrophages residing in atherosclerotic plaques (Sanchez-Gaytan et al., 2015). Although using PLGA core in this system enables loading of different therapeutic and/or imaging molecules, it can impose limitations on ApoA-I conformational flexibility resulting in impaired cholesterol efflux (Tall et al., 2008; Tang et al., 2016). Moreover, this artificial polymeric core induced changes in the shape of these nanoparticles to be more spherical rather than discoidal shape of native HDL, and their sizes increased significantly in comparison to the HDL.

In addition to the nanoparticle size and shape, the phospholipid core was studied and 1-palmitoyl-2-oleoyl-sn-glycero-3-phosphocholine based HDL nanoparticle showed the highest similarity to natural HDL (Tang et al., 2016). Such HDL like nanoparticles have been used to deliver statins into inflammatory atherosclerotic plaques. For instance, reconstituted HDL (rHDL) nanoparticles loaded with simvastatin [S]-rHDL have been designed and successfully employed to deliver the payload into inflammatory plaques in ApoE knockout mice (Duivenvoorden et al., 2014). A comparison was made for low doses of [S]-rHDL (15 mg/kg simvastatin, 10 mg/kg ApoA-I) that was administered intravenously biweekly for 12 weeks vs. orally administered simvastatin (15 mg/kg per day) and the results showed lower plaque macrophage content for the [S]-rHDL group. Interestingly, delivery of a high dose (60 mg/kg simvastatin, 40 mg/kg ApoA-I) by this nanosystem into ApoE knockout mice in four intravenous injections showed significant mitigation effects on plaque inflammation while rHDL alone could not decrease the sizes of plaque area based on histological analysis. The stronger effect for short term high [S]-rHDL group indicated the importance of delivering higher amounts of simvastatin for inhibiting atherosclerotic plaque inflammation.

Although rHDL is an attractive drug delivery system for cardiovascular diseases, undesired drug leakage is a drawback of this system. One reason for this leakage is believed to be the interaction of drug loaded rHDL with the lecithin cholesterol acyltransferase that can remodel this particle (Zhang et al., 2013). An approach to decrease r-HDL reactivity with the lecithin cholesterol acyltransferase is through the insertion of arachidonic acid into the phospholipid bilayers of rHDL (He et al., 2014). Such adjustment of physicochemical properties of rHDL could enhance nanocarrier design for drug delivery to atherosclerotic plaques (He et al., 2015).

In order to improve drug delivery property of HDL like particles, dextran sulfate (DXS) has been used to coat rHDL as DXS can bind with scavenge receptor AI highly expressed on activated macrophages and foam cells in inflammatory plaques through electrostatic interactions (De Winther et al., 2000; Chao et al., 2012). In this design, atorvastatin was loaded in a spherical PLGA core encapsulated in liposomes. Then, it was decorated by ApoA-I and finally coated with DXS (AT-DXS-LP-rHDL) (Zhao et al., 2017). Cellular study showed the ability of these particles to facilitate cholesterol efflux from macrophages (RAW264.7 and THP-1 cells). Moreover, these particles were able to reduce TNFα and IL-6 production in macrophages due to atorvastatin release. It is noteworthy that ApoA-I and DXS coating both helped retard atorvastatin release. In addition, ox-LDL uptake by macrophages was significantly reduced because high affinity DXS competes with ox-LDL for SR-AI binding.

Since the incorporation of ApoA-1 into a small molecule therapeutic agent can be challenging due to the hydrophobicity and large size (21–31 kDa) of ApoA-1, a mimetic peptide containing 18-amino acids, known as “4F” was employed by Mansukhani and coworkers to prepare peptide amphiphile nanofibers loaded with liver X receptor agonist GW3965 (LXR) (Mansukhani et al., 2019). Intravenous administration of this formulation (8-weeks, two injections per week) to ApoE knockout mice showed therapeutic effects comparable to the free drug (LXR) while the liver toxicity was reduced. Future work on this formulation would focus on physicochemical analysis on peptide amphiphile and covalently loading LXR to improve the overall therapeutic efficacy.

## Blood Cells as Trojan Horses for Anti-Atherosclerosis Drug Delivery

Harnessing blood cells for drug delivery has important features rendering it attractive for atherosclerosis therapy. For instance, the blood circulation time of nanomedicine will be increased due to the inherent abilities of cellular membrane enabling the cells to evade the phagocytic system. Accordingly, Wang and coworkers packed rapamycin (RAP) loaded PLGA nanoparticles in the membrane of red blood cells (RBC) termed RBC/RAP@PLGA (Wang et al., 2019). RAP is an immunosuppressant molecule with anti-atherogenic effects (Waksman et al., 2003; Castro et al., 2004). This formulation showed a significantly higher therapeutic effect in ApoE knockout mice following a 1-month treatment compared to the RAP@PLG nanoparticles lacking the RBC membrane. Similarly, Song and coworkers introduced RAP loaded PLGA nanoparticle coated with platelet membrane named RAP-PNP (Song et al., 2019). Platelets play important roles in the pathogenesis of atherosclerosis and they have inherent affinities to home to the plaques (Lindemann et al., 2007). Four-week treatment of ApoE knockout mice with RAP-PNP led to superior therapeutic effects compared to free RAP or RAP-NP with a favorite safety profile for long-term use. In a similar study, RAP loaded nanoparticles coated by macrophage membrane (MM), i.e., the MM/RAP NPs, showed an efficient accumulation in atherosclerotic plaques in ApoE knockout mice and inhibited the progression of disease (Wang et al., 2021). Moreover, MM/RAP NPs had an enhanced blood retention time compared to uncoated RAP NPs indicating the importance of MM coating to improve pharmacokinetics of the formulation.

Neutrophils can be recruited to atherosclerotic plaques through their interactions with ICAM-1, E-selectin, P-selectin, CXCL1/KC, CXCL2 and CXCL4 (Baetta and Corsini, 2010; Williams et al., 2011; Soehnlein, 2012; Kolaczkowska and Kubes, 2013). Xue and coworkers introduced cellular vehicles based on neutrophils as a novel strategy for targeting atherosclerotic plaques (Xue et al., 2019), which can be a potential strategy for delivering cargoes. Although this work showed excellent targeting properties of engineered neutrophils into the plaques in the ApoE knockout mouse model, the encapsulation of an anti-atherosclerotic compound for therapy has yet to be evaluated.

## HA Based Nanoparticles Targeting CD44 for Atherosclerosis Treatment

Overexpressed CD44 in atherosclerotic plaques is an important target for drug delivery. HA is a naturally occurring polysaccharide with high affinity for CD44, which has been used to decorate rHDL for targeting inflammatory atherosclerotic plaques (Slevin et al., 2007). Simvastatin loaded PLGA nanoparticles have been encapsulated into liposomes and decorated with ApoA-I and HA (ST-HA-PLGA-rHDL). HA immobilization was carried out by two different strategies. The first strategy includes covalent conjugation of HA onto available amine groups of ApoA-I through 1-ethyl-3-(3-dimethylaminopropyl) carbodiimide (EDC) and *N*-hydroxy-succinimide (NHS) coupling agents (ST-HA-(C)-PLGA-rHDL). The second strategy was electrostatic absorption of HA by cationic ST-PLGA-rHDL (ST-HA-(E)-PLGA-rHDL). Testing these nanostructures *in vivo* on a rabbit model of atherosclerosis indicated better atheroprotective activity for ST-HA-(C)-PLGA-rHDL. This observation has been explained through a better shielding effect of covalent HA coating that decreases their liver uptake (Zhang M. et al., 2017). In addition, HA coating enhanced nanoparticle accumulation in atherosclerotic plaques due to its interaction with CD44. This effect has been shown in the rabbit model of atherosclerosis through quantification of DiR labeled HA-(C)-PLGA-rHDL in the aortic tree.

Huang and coworkers recently introduced HA conjugated atorvastatin nanoparticle (HA-ATV-NPs) capable of delivering atorvastatin into the inflammatory atherosclerotic plaques in ApoE knockout mice (Hossaini Nasr et al., 2020). HA-ATV-NPs were constructed through chemical conjugation of ATV to HA backbone, which enabled the loading of a significant amount of ATV (35% by weight) into the NPs rendering it attractive for statin therapy. The therapeutic effects by HA-ATV-NPs were observed following a 1-week treatment (8.5 mg ATV.kg^−1^, one injection every other day). Moreover, the therapeutic effect could be monitored non-invasively by magnetic resonance imaging aided by a superparamagnetic iron oxide based nanoworms bearing HA (Hossaini Nasr et al., 2018), highlighting the utility of HA for plaque targeting.

HA has been utilized to reduce rHDL uptake by liver’s scavenger receptor class B type I (SR-BI). Lovastatin loaded r-HDL (LT-rHDL) has been decorated with HA, which facilitated their interactions with up-regulated CD44 receptors in inflammatory plaques while reducing their liver uptake (Liu et al., 2014). Functionalizing the Apo proteins with HA helped LT-rHDL escape from the reticuloendothelial system (RES) and improved lovastatin circulation time *in vivo*.

**FIGURE 5:**
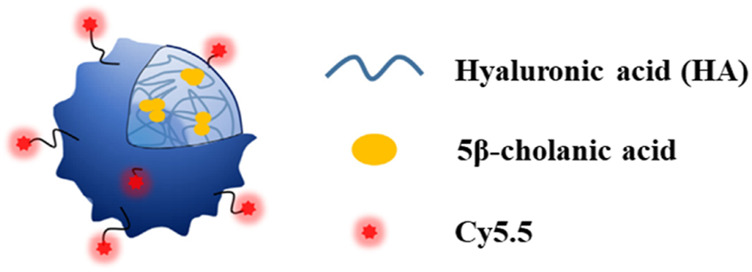
Conjugation of hydrophobic 5-cholanic acid with hyaluronan resulted in self-assembled nanoparticle formation. This formulation could be a drug carrier for atherosclerotic plaques imaging and/or therapy (Copyright permission from Elsevier).

In addition to HA-rHDL, another type of HA nanoparticle has been prepared by conjugation of hydrophobic 5β-cholanic acid to HA through NHS, EDC chemistry *via* a diamine linker. The resulting conjugate can self-assemble to nanoparticles in water due to the hydrophilic nature of HA backbone and hydrophobic property of 5β-cholanic acid (Figure 5). Labeling this NP system with fluorescent molecules such as Cy5.5 and fluorescein isothiocyanate demonstrated that this HA-NP could be selectively taken up by CD44 expressing cells *in vitro* through receptor mediated endocytosis. Moreover, injection of this nanoparticle to ApoE knockout mice showed its accumulation in atherosclerotic plaques and further immunohistochemistry studies confirmed co-localization of HA-NPs with CD44 and another HA receptor, i.e., hyaluronic acid receptor for endocytosis (HARE) in atherosclerotic lesions (Lee et al., 2015).

In nature, HA can vary significantly in length, and its molecular weight can significantly impact its biological activities. For example, high molecular weight HA (megadaltons, MDa) can suppress inflammation and angiogenesis, while low molecular HA oligomers are inflammatory and angiogenic (Rooney et al., 1995; Masuko et al., 2009). Subsequently, in addition to their CD44 targeting property, HA atheroprotective effect has been investigated through PET/MRI. HA (MW 66–99 kDa) has been used to prepare radiolabeled HA-NP (^89^Zr-HA-NP) and administered to ApoE knockout mouse and rabbit models of atherosclerosis (Beldman et al., 2017). HA-NP inhibited the production of TNFα, IL-2, IL-6 and nitric oxide by macrophages *in vitro*. Furthermore, HA-NP treatment stabilized plaques *in vivo*, which increased collagen content of plaques up to 30–40% while reducing macrophage content by 30% in comparison with control groups. One hypothesis to explain these observations is that aggregation of HA polymer to form HA-NPs might imitate the anti-inflammatory effect of high molecular weight (MDa) HA.

## Solid Lipid Nanoparticles and Nanofibers for Atherosclerosis Treatment

Methotrexate (MTX) is an immunomodulatory drug that is often used for chronic inflammatory disease (Chan and Cronstein, 2002) and its atheroprotective effect in the presence of systemic inflammation has been the subject of several studies (Bulgarelli et al., 2012; Ronda et al., 2015). MTX can decrease inflammatory cytokine production and downregulate the expression of adhesion molecules although the specific mechanism is not fully understood (Chan and Cronstein, 2002; Gerards et al., 2003). MTX has been formulated in solid lipid nanoparticle (MTX SPN) for targeted delivery to atherosclerotic plaques. In this formulation, MTX has been loaded into the hydrophobic PLGA core and decorated with phospholipids, which were found to decrease inflammatory cytokines production (IL-6 and TNFα) upon *in vitro* incubation with J774.A1 macrophages. Moreover, MTX SPN has been labeled with DiD and rhodamine for fluorescent imaging and radiolabeled with ^64^Cu for further *in vivo* studies. These particles were taken up by macrophages releasing their cargo inside the cells. In addition, circulating monocytes can take up them and infiltrate into atherosclerotic plaques. *In vivo* evaluation of MTX SPN on ApoE knockout mice showed 50% reduction of plaque burden following biweekly administration of 1 mg/kg MTX in 1 month (Stigliano et al., 2017).

As discussed earlier, macrophage targeting is a known strategy for atherosclerosis therapy and/or imaging. Lipid-latex (LiLa) hybrid nanoparticles have been designed for targeting inflammatory macrophages. In this design, a hydrophobic polymer latex serves as a drug reservoir, while lipids bring colloidal stability to the system as well as targeting properties. Phosphatidylserine and cholesterol-9-carboxynonanoate have been used as lipid coating. These molecules served as “eat me” signal for macrophages enhancing macrophage uptake (Poon et al., 2014). In addition, over-expression of phospholipase A2 (Goncalves et al., 2012) in inflammatory macrophages could disrupt lipid coating and trigger drug release inside the macrophages (Bagalkot et al., 2015). Testing LiLa NP for imaging atherosclerotic plaques in ApoE knockout mice showed their accumulation in plaques while control bare latex nanoparticle mostly accumulated in the liver. However, LiLa NP may not be a good candidate for clinical translation to humans due to the poor biodegradability properties of polystyrene latex.

The immune system can be regulated to reduce the plaque burden. Accordingly, Flores and coworkers developed single-walled carbon nanotubes (SWNTs) loaded with SHP-1 inhibitor termed as SWNT-SHP1i (Flores et al., 2020). SHP-1i is a small molecule inhibitor of CD47’s downstream effector molecule, resulting in phagocytic function recovery that was suppressed in the plaques. It is important to note that the impairment of phagocytic activity in inflammatory sites contribute to the pathology of atherosclerosis. SWNT-SHP1i was able to restore the phagocytic activity of macrophages in the plaques, resulting in plaque expansion inhibition and reduction of inflammation.

## Administration Routes of Atheroprotective Nanomedicine

Route of nanoparticle administration is an important consideration. While intravenous (iv) administration of nanoparticles is commonly utilized, formulations for oral delivery of nanoparticles have been investigated. For example, rosuvastatin was incorporated into solid lipid nanoparticles for oral administration and it led to not only better bioavailability of drug, but also higher decrease of plasma cholesterol and LDL levels compared with the free drug (Beg et al., 2017). However, these rosuvastatin loaded solid lipid nanoparticles have only been investigated under hyperlipidemia-like condition, not in atherosclerotic models.

In another study for oral delivery, RAP loaded nanoparticles have been prepared through self-assembly of cationic polymer polyethyleneimine. These positively charged nanoparticles have been packed into microcapsule obtained from yeast *Saccharomyces cerevisiae* to form complex yeast-derived microcapsule (YC) (Zhang X. et al., 2017). Through the transcytosis mechanism, the YC could be absorbed by M cells in Peyer’s patches, which are aggregated lymphoid follicles in the gut (Jung et al., 2010). Following the absorption, monocytes and/or macrophages are able to uptake them by endocytosis and relocate them in inflammatory atherosclerotic plaques. In addition to RAP, this nanoparticle system has been tuned to deliver other drugs such as ursodeoxycholic acid and sulindac to atherosclerotic plaques. Using an oral formulation that can target inflammatory atherosclerotic plaques efficiently is much more convenient than iv injections, which is advantageous for future clinical translation.

Intraperitoneal (ip) injection of nanoparticle is another common route of administration for atheroprotective nanoparticles (Guo et al., 2018). Interestingly, superiority of ip over iv injection of an HDL like nanoparticle was reported by Jung and coworkers (Jung et al., 2014). However, other HDL like nanoparticles reported in the same work were administered iv, indicating that individual characteristic of HDL like nanoparticles, such as size and surface charge, likely dictated the best route of their administration. Finally, subcutaneous (sc) injection of hydrogel has been studied for sustained delivery of anti-inflammatory nanoparticles (Yi et al., 2020). In another study, insulin-like growth factor (IGF)-1 was formulated in nanofibers/hydrogel showed atherosclerosis inhibition *in vivo* following sc administration in ApoE knockout mice (Shang et al., 2020). In general, most nanoparticles designed for active targeting of atherosclerosis have been administered iv and other routes of administration take advantage of the abilities of macrophages to take up the particles and traffic to the inflammatory sites including atherosclerotic plaques.

## Conclusion and Future Directions

Increasing and improving the stability of a rupture prone plaque should be the first high priority target for an atheroprotective nanoparticle. Obviously, this is the main reason why the majority of the research in this area focuses on inflammatory aspect of atherosclerosis, the major contributor of plaque instability. Another key step towards management of inflammatory atherosclerotic plaques is the diagnosis of unstable plaques by imaging. Therefore, designing of imaging probes for detection of unstable plaques is important (Vazquez-Prada et al., 2021). One of the future directions for this field would be to develop theranostic nanoparticle that not only impose therapeutic effects on plaques but also help clinicians to monitor the treatment progress. It is noteworthy to mention that a lack of proper animal models resembling the unstable plaque features with human is an important limitation factor for the field. Finally, another challenge that has not been fully addressed yet, is adjusting pharmacokinetic of nanoparticles targeting atherosclerosis. Therefore, optimizing the blood circulation time, minimizing the liver uptake through elaborated physicochemical design, and introducing specified ligands targeting unstable plaques will introduce more nanomedicine candidates to the clinical trials in the future.

Targeting of inflammatory atherosclerosis plaques is highly desired for delivering therapeutic molecules to the sites of action. To achieve this goal, a multitask nanocarrier should be designed with the capability of reaching atherosclerosis plaques selectively and releasing its payload. Although, HDL, the naturally occurring nanoparticle, has inspired scientists to fabricate HDL mimetic nanoparticles, the challenges have not been fully addressed due to the high affinity of liver for these particles as well as the high cost of ApoA (1 mg costs $333 from Sigma-Aldrich). In addition, physicochemical properties of these nanoparticles should be carefully tuned to get the highest HDL like activity. Therefore, researchers have investigated other nanocarriers to deliver anti-inflammatory or anti-proliferative molecules. Among anti-inflammatory therapeutics, statins have been investigated the most due to their applications in the clinic for management of atherosclerosis. With suitably designed nanoparticle systems, significant therapeutic effects towards atherosclerotic plaques can be achieved to reduce the adverse impacts of atherosclerosis.
